# Effect of photodynamic therapy mediated by hematoporphyrin derivatives on small cell lung cancer H446 cells and bronchial epithelial BEAS-2B cells

**DOI:** 10.1007/s10103-024-04013-2

**Published:** 2024-02-17

**Authors:** Cunzhi Lin, Yuanyuan Zhang, Jiemei Liao, Shichao Cui, Zhe Gao, Weizhong Han

**Affiliations:** 1 Department of Respiratory and Critical Care Medicine, The Affiliated Hospital of Qingdao University, Qingdao, 266003 China; 2 Department of Respiratory and Critical Care Medicine, Yantai Yuhuangding Hospital, Yan Tai, 264001 China; 3 Department of Respiratory and Critical Care Medicine, Xiangtan Central Hospital, Xiangtan, 411199 China

**Keywords:** Photodynamic therapy, Hematoporphyrin derivatives, Lung cancer, H446 cells, BEAS-2B cells

## Abstract

To investigate the effects of photodynamic therapy (PDT) mediated by hematoporphyrin derivatives (HPD) on the proliferation of small cell lung cancer H446 cells and bronchial epithelial BEAS-2B cells. H446 cells and BEAS-2B cells were cultured in vitro with different concentrations of HPD(0, 5, 10, 12, 15, 20 μg/mL) for 4 h, and then irradiated with 630 nm laser with different energy densities (0, 25, 50, 75, 100 mW/cm2). Cell viability of H446 cells and BEAS-2B cells were detected by CCK8 assay. The cell apoptosis was observed with Annexin V-FTTC/PI double staining and Hoechst 33258. The RT-PCR examination was applied to detect the transcriptional changes of the mRNA of Bax、Bcl-2, and Caspase-9. The results of CCK8 showed that when the HPD was 15 μg/mL and the laser power density reached 50 mW/cm2, the cell viability was significantly decreased compared with the black control group. Hoechst 33258 staining showed that with the increase of HPD concentration, the cell density was reduced, and apoptotic cells increased. Flow cytometry assay revealed that the apoptotic rates of the HPD-PDT group of H446 cells and BEAS-2B cells were significantly different from those of the blank control group. The RT-PCR examination showed that the expression levels of Bax and Caspase-9 mRNA in the HPD-PDT group were up-regulated, while the expression levels of Bcl-2 mRNA were down-regulated significantly. HPD-PDT can inhibit H446 cells and BEAS-2B cells growth. The mechanism may be related to up-regulating the expression levels of Bax and Caspase-9 mRNA and down-regulating the expression levels of Bcl-2 mRNA.

## Introduction

The occurrence of malignant tumors is becoming more and more common. Lung cancer is the most frequent cause of mortality worldwide [1]. Although our understanding of the risk factors, development and treatment of lung cancer has improved, due to the increasingly serious environmental pollution and the massive consumption of tobacco, lung cancer continues to be the world's leading cause of cancer death [2, 3]. According to its pathological characteristics, the World Health Organization divides lung cancer into small cell lung cancer (SCLC) and non-small lung cancer (NSCLC), and SCLC accounts for 15% of lung cancer [4, 5]. SCLC is highly malignant, aggressive and easy to metastasize early [6]. Most of the patients with SCLC are diagnosed at an advanced stage and have lost the best time for surgical treatment. Radiotherapy and chemotherapy have become the main treatment. Although SCLC is sensitive to radiotherapy and chemotherapy in the early stage, some patients will develop tumor resistance and disease progression after treatment for a period of time, and the serious side effects are inevitable [7]. Despite a large number of clinical trials, the systemic treatment of SCLC has not changed significantly. The 5-year survival rate of SCLC is usually less than 7% [8]. Therefore, it is very necessary to explore new SCLC treatment methods.

Photodynamic therapy (PDT) is a modern and non-invasive form of therapy. It consists of three essential components: photosensitizer, light and oxygen [9]. None of the components is individually toxic, but they can initiate a photochemical reaction that leads to cell death via apoptosis or necrosis [10]. PDT shows unique therapeutic advantages in cancer treatment due to its selective killing of tumor cells and less trauma. It is a new adjuvant therapy for malignant tumors [11]. Clinical trials conducted by Bown et al. showed that PDT can produce necrosis in pancreatic cancers and effectively prolong the survival time of patients [12]. Early experiments by Santos et al. proved that Photodynamic therapy mediated by methylene blue has a significant killing effect on breast cancer cells and could use as a powerful adjunct therapy to breast tumors [13]. Moreover, studies have reported that PDT also has therapeutic effects on basal cell carcinoma, bladder cancer, colon cancer, and so on [14–16].

In the 1970s, KELLY [17]et al. successfully applied Hematoporphyrin Derivative ( HPD) as photosensitizer to treat bladder cancer, thus pioneering the PDT in tumor treatment and HPD is also the first photosensitizer used in clinic for the PDT [10]. With the development and maturity of pulmonary interventional therapy, PDT is gradually being applied to the treatment of lung cancer. In recent years, Hematoporphyrin derivative (HPD) has been used as a photosensitizer in the PDT of lung cancer, it could selectively accumulate in malignant tumors [18, 19]. Although as the representative of the first generation photosensitizer, it has many shortcomings such as strong light toxicity but at present PDT for lung cancer is still using HPD. Photodynamic therapy mediated by Hematoporphyrin derivatives (HPD-PDT) has been shown to exhibit an antiproliferative effect on lung adenocarcinoma A549 cells [20].

This study aimed to investigate the proliferation inhibition effect of HPD-PDT on H446 cells and BEAS-2B cells and explore the changes of apoptosis-related factors involved.

## Materials and methods

### Materials

Human small cell lung cancer H446 cell and bronchial epithelial BEAS-2B cell lines were obtained from the American Type Culture Collection. The Dulbecco’s modified Eagle’s medium (DMEM) were purchased from Hyclone. The CCK8 kit was from Med ChemExpress. Hoechst 33,258 dyeing liquid, cell cycle, and apoptosis detection kit were obtained from Beyotime. Hematoporphyrin derivative was purchased from Huading Modern Biologic& Chemical Co. Ltd. The reverse transcription kit and SYBR Green fluorescent quantitative PCR kit were from TaKaRa. LED-IB photodynamic therapy instrument was made by Wuhan Yage Photoelectric Technology Co. LTD (China). Mode of operation: continuous. The output wavelength was: Red light 630 nm; Output power: 80 mW/cm^2^.

### Cell culture

Human small cell lung cancer H446 cells and bronchial epithelial BEAS-2B cells were cultured in DMEM supplemented with 10% fetal bovine serum, 100 U/ml penicillin, and 100 mg/ml streptomycin in a humidified atmosphere containing 95% air and 5% CO_2_ at a constant temperature of 37˚C. Cells in the logarithmic phase were used in the experiment.

### Photodynamic therapy mediated by hematoporphyrin derivatives

H446 cells and BEAS-2B cells were randomly divided into four groups and treated as follows: In HPD-PDT group, cells were incubated with various concentrations of HPD (5, 10, 12, 15, 20 μg/mL) for 4 h respectively, then cells were exposed to 630 nm laser with a different energy density of 25, 50, 75, 100 MW/cm^2^. for 2 min. In photosensitizer group, cells were treated with HPD. In irradiation group, cells were only received light exposure with 630 nm. In blank control group, cells were treated without HPD and light irradiation. The distance of the light source away from samples was 2 cm. Cells were incubated for 24 h with fresh medium before assays were performed.

### CCK8 assay

The status of cell growth was determined by Cell Counting Kit-8 reagent. H446 cells and BEAS-2B cells were seeded in 96-well plates at a density of 1 × 10^4^ cells per well. After supernatant was discharged, 0, 5, 10, 12, 15 and 20 μg/mL HPD was added respectively, 3 wells in each concentration group. After 4 h of incubation in a dark environment, the medium was replaced with serum-free DMEM. Then H446 cells and BEAS-2B cells were exposed to 630 nm red laser red with different energy densities of 0, 25, 50, 75 and 100 MW/cm^2^ for 2 min respectively. The supernatant was removed after being incubated for 24 h and the culture medium containing 10% CCK8 was added to each well. The absorbance values at 450 nm were measured using an automatic enzyme marker.

### Hoechst33258 staining

The best concentration of photosensitizer and power density of laser were screened out by the CCK8 assay for follow-up experiments. The cell slides were soaked in 75% ethanol and rinsed with phosphate-buffered saline (PBS). Then put the cell slides in 12-well plates and inoculated with H446 cells and BEAS-2B cells respectively. After being cultured for 24 h, cells were randomly divided into a blank control group, pure photosensitizer group (15 μg/mL HPD incubation, without laser), pure light group (50 MW/cm^2^ laser irradiation, without photosensitizer incubation), and HPD-PDT group (5, 10, 12, 15, 20 μg/mL incubated with the concentration of photosensitizer, 50 MW/cm^2^ laser). After the treatment, cultured the cells in the incubator for 24 h. Then the supernatant was removed and cells were incubated with 0.5 mL fixative for 15 min. The cells were stained with 0.5 mL of Hoechst 33,258 for 5 min after abandoning the fixative and washing the cell slides three times with PBS. The cells were washed again and antifluorescence quenching sealing liquid was dropped on the slides, covered with a stained glass cover and incubated at room temperature for 15 min. The cells were observed under an inverted fluorescence microscope (Olympus Co. Japan) and photographed.

### Flow cytometry

The cells were seeded in 6-well plates and cultured for 24 h. After cells were serum-starved, they were divided into blank control group, pure photosensitizer group (15 μg/mL photosensitizer incubation), pure light group (50 MW/cm^2^ laser irradiation), and HPD-PDT group (15 μg/mL photosensitizer incubation, 50 MW/cm^2^ laser). The cells were collected and washed twice with ice-cold PBS, and resuspended in 500 μL Binding Buffer. Then added 5 μL AnnexinV-FITC and 5 μL PI staining agent sequentially and incubated in dark for 15 min. Finally, the apoptotic rate was assayed by flow cytometer.

### Reverse transcription-polymerase chain reaction (RT-PCR)

Total RNA was extracted from the cells using Trizol reagent according to the manufacturer’s instructions and concentration was measured by microplate reader. Single-stranded cDNA was acquired by reverse transcription with oligo(dT) primer according to the manufacturer's instructions. The reverse transcription conditions were 10 min at 95 °C, 5 s at 95 °C for 40 cycles, and annealing at 60 °C for 30 s followed by an extension at 60 °C for 3 min in a 20 µL reaction mixture. Then the cDNA was used for PCR amplification, and the Gene expression was performed by RT-PCR, measured by the SYBR Green PCR master mix. Primers for Bax, Bcl-2, Survivin, Caspase-3 and GAPDH gene were designed. Real time-PCR was followed by melting curve analysis with the following cycling program: initial activation at 95 °C for 10 min, followed by 40 cycles of denaturation at 95 ℃ for 5 s, annealing at 60 °C for 30 s, and extension at 60 °C for 3 min. The relative expression of genes in each group were calculated by 2–ΔΔCt with β-actin as the internal reference gene. Results are expressed as mean ± standard deviation.

### Statistical analysis

SPSS23.0 statistical software was used for statistical analysis. Each group of experiments above was repeated 3 times independently, and the experimental data were expressed as mean ± standard deviation (x±s). The overall data of multiple groups were compared by single factor analysis of variance. The pairwise comparison between groups was analyzed by LSD-t test. P≤0.05 suggested that the difference was statistically significant.

## Result

### HPD-PDT inhibited the growth of H446 cells and BEAS-2B cells

**Fig. 1:**
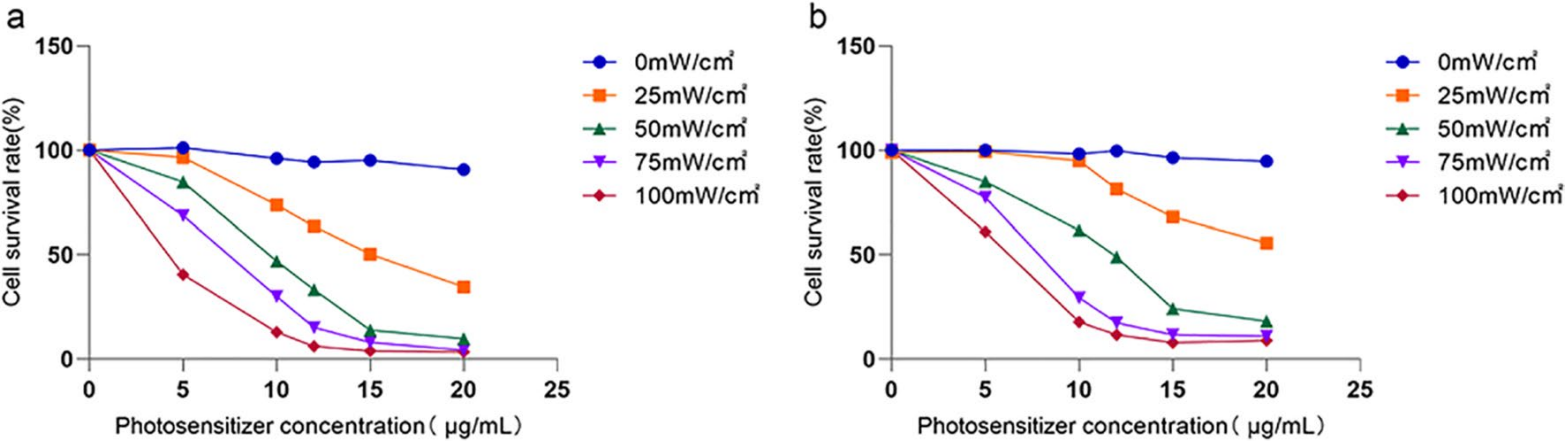
Effects of hematoporphyrin derivatives mediated photodynamic therapy on H446 cells and BEAS-2B cells. **a**: Viability of H446 cells measured with CCK8; **b**: Viability of BEAS-2B cells measured with CCK8

The photosensitizer alone or laser irradiation has no obvious killing effect on H446 cells and BEAS-2B cells. The killing effect of HPD-PDT increases with the increase of the HPD concentration and laser intensity (Fig. 1). Compared with the control group, 15 μg/ml HPD combined with 50 MW/cm^2^ laser irradiation can significantly inhibit the growth of H446 cells and the cell survival rate was 13.96% (*p* < 0.0 5; Fig. 1a). When HPD concentration was raised to 15 μg/mL and laser intensity raised to 50 MW/cm^2^, the viability of BEAS-2B cells was decreased significantly compared with the control group and the result was 24.23% (*p* < 0.0 5; Fig. 1b). The survival rate of H446 cells was lower than that of BEAS-2B cells, and the difference was statistically significant. Increasing the concentration of HPD or laser intensity did not significantly increase the severity of growth inhibition. As a result, we chose 15 μg/mL of HPD with 50 MW/cm^2^ laser irradiation for the subsequent experiments.

### HPD-PDT induced the apoptosis of H446 cells and BEAS-2B cells

**Fig. 2:**
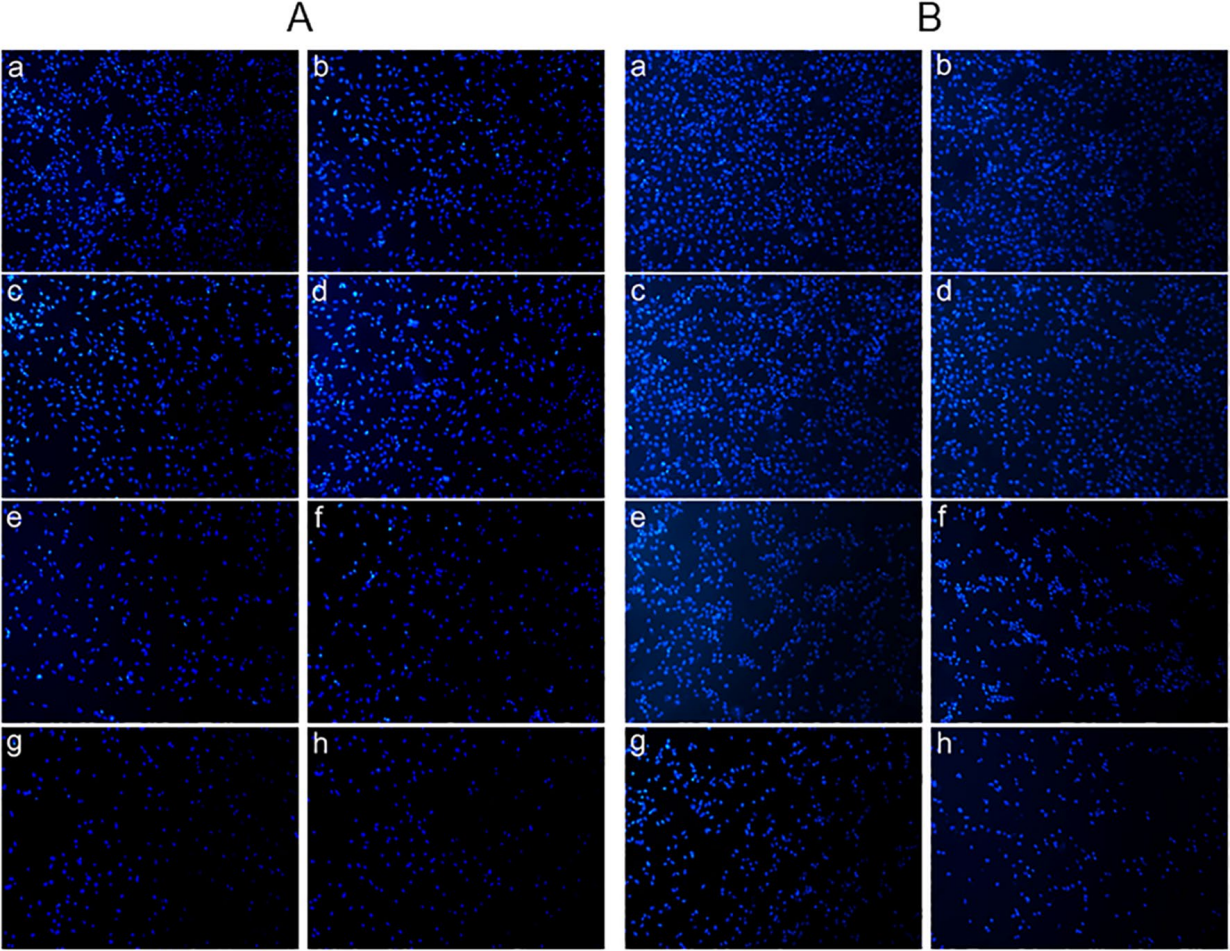
Effect of hematoporphyrin derivatives mediated photodynamic therapy on apoptosis of H446 cells and BEAS-2B cells. Panel **A**: Apoptotic morphological changes of H446 cells observed by inverted fluorescence microscope with Hoechst 33,258 staining (× 40). Panel **B**: Apoptotic morphological changes of BEAS-2B cells observed by inverted fluorescence microscope with Hoechst 33,258 staining (× 40). Graphs **a**, **b**, **c**, **d**, **e**, **f**, **g**, and h in each panel represent black control group, pure photosensitizer group (15 μg/mL), irradiation group(50 MW/cm^2^), 5 μg/mL HPD-PDT group, 10 μg/mL HPD-PDT group, 12 μg/mL HPD-PDT group, 15 μg/mL HPD-PDT group, 20 μg/mL HPD-PDT group respectively

**Fig. 3:**
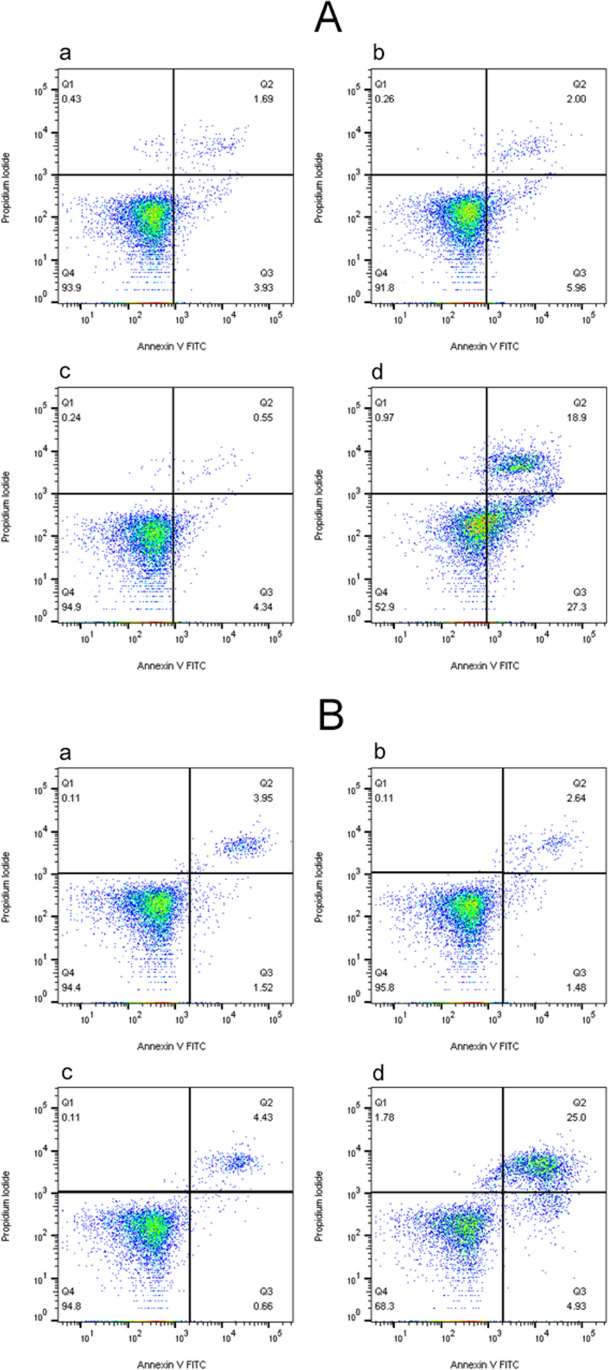
Effect of hematoporphyrin derivatives mediated photodynamic therapy on apoptosis of H446 cells and BEAS-2B cells. Panel A: Apoptotic effects of hematoporphyrin derivative-mediated photodynamic therapy on H446 cells with flow cytometry. Panel B: Apoptotic effects of hematoporphyrin derivative-mediated photodynamic therapy on BEAS-2B cells with flow cytometry. Graphs **a**, **b**, **c** and d in each panel represent black control group, pure photosensitizer group (15 μg/mL), irradiation group(50 MW/cm^2^), HPD-PDT group (15 μg/mL + 50 MW/cm^2^) respectively

As illustrated in Fig. 2, after cells were treated as the above methods, apoptotic cells with chromatin condensation and hyperchromatic nuclei were seen in HPD-PDT groups of both H446 cells and BEAS-2B cells by Hoechst 33258 staining. With the increase of HPD concentration, the cell density was reduced and apoptotic cells increased. However, there were no obvious apoptotic cells in control group (Fig. 2a, A and 2a, B), simple photosensitizer group (Fig. 2b, A and 2b, B) and laser irradiation group (Fig. 2b, A and 2b, B) of two cells. Quantitative apoptotic analysis was done using Flow cytometry assay. The apoptosis rates of control group, simple photosensitizer group, irradiation group, HPD-PDT group in H446 cells were (5.99 ± 0.32) %, (6.55 ± 1.40) %, (5.20 ± 0.68) %, and (46.73 ± 1.38) %, respectively (Fig. 3a). There were no significant differences between the black control group, simple photosensitizer group and irradiation group (all* P* > 0.05). Compared with the control group, The apoptosis rate of the HPD-PDT was significantly increased. The results of BEAS-2B cells were (5.69 ± 0.30) %, (4.69 ± 0.50) %, (5.30 ± 0.48) %, and (28.56 ± 1.80) %, respectively (Fig. 3b). The apoptosis rate of HPD-PDT group was significantly higher than that of the other three groups (all *P* < 0.05).

### HPD-PDT increased the mRNA expression levels of Bax and Caspase-9, and decrease the mRNA expression levels of Bcl-2

**Fig. 4:**
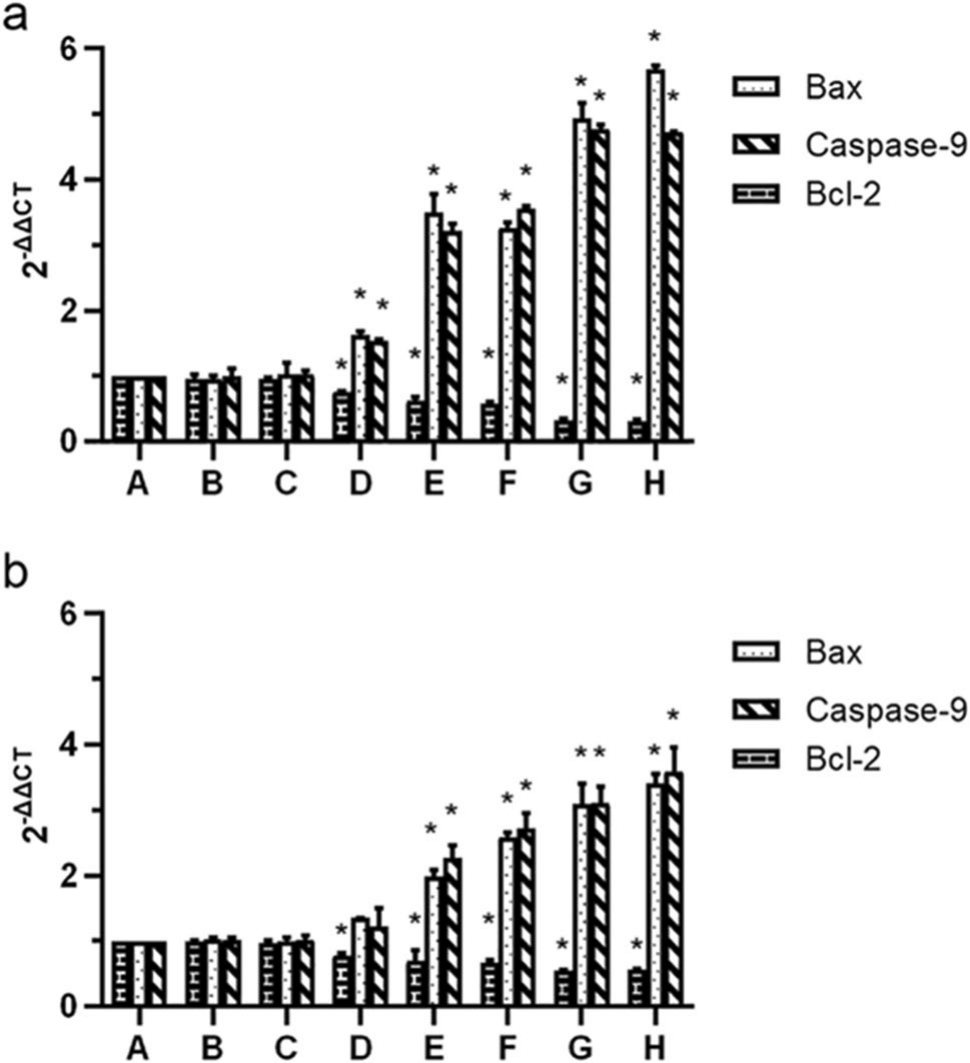
Effects of hematoporphyrin derivative-mediated photodynamic therapy on Bax, Caspase-9 and Bcl-2. a: The mRNA expression level of Bax, Caspase-9 and Bcl-2 in H446 cells after HPD-PDT. b: The mRNA expression level of Bax, Caspase-9 and Bcl-2 in H446 cells after HPD-PDT. (**A**) control group (**B**) pure photosensitizer group (15 μg/mL) (**C**) irradiation group(50 MW/cm^2^) (**D**) 5 μg/mL HPD-PDT group (**E**) 10 μg/mL HPD-PDT group (**F**) 12 μg/mL HPD-PDT group (**G**) 15 μg/mL HPD-PDT group (**H**) 20 μg/mL HPD-PDT group. Data are presented as mean ± SD of three independent experiments. *P < 0.05 compared with the control group

Compared with control group, the relative mRNA expression levels of Bax of 5, 10, 12 15, and 20 μg/ml HPD-PDT group in H446 cells were significantly increased to 1.625 ± 0.056, 3.490 ± 0.290, 3.261 ± 0.079, 4.928 ± 0.239 and 5.677 ± 0.059, respectively. The results of Bcl-2 of 5, 10, 12 15, and 20 μg/ml HPD-PDT group in H446 cells were significantly decreased to 0.761 ± 0.011, 0.611 ± 0.063, 0.582 ± 0.014, 0.322 ± 0.028 and 0.303 ± 0.032, respectively. Those of Caspase-9 were 1.527 ± 0.037, 3.217 ± 0.111, 4.774 ± 0.066 and 4.724 ± 0.016, respectively. The differences between the HPD-PDT groups and the control group were statistically significant (all *P* < 0.05; Fig. 4a). Figure 4b indicated that the expression of Bax mRNA in 10, 12 15, and 20 μg/ml HPD-PDT group were significantly higher than in control group of BEAS-2B cells with the results of 1.988 ± 0.096, 2.585 ± 0.072, 3.095 ± 0.311 and 3.413 ± 0.142, and those of Caspase-9 were higher than in control group of BEAS-2B cells with the results of 2.263 ± 0.188, 2.711 ± 0.244, 3.102 ± 0.247 and 3.592 ± 0.361. The expression of Bcl-2 mRNA in 5, 10, 12 15, and 20 μg/ml HPD-PDT group were significantly lower than in control group of BEAS-2B cells with the results of 0.777 ± 0.040, 0.690 ± 0.157, 0.667 ± 0.039, 0.545 ± 0.017 and 0.565 ± 0.006. The differences between the HPD-PDT groups and control group were statistically significant. There was no significant difference between the relative mRNA expression levels of Bax, Caspase-9, and Bcl-2 in control group, simple photosensitizer group, and irradiation group of H446 cells and BEAS-2B cells (Fig. 4).

## Discussion

PDT is a clinically approved minimally invasive treatment that can selectively exert targeted cytotoxic activity on malignant cells [21]. It is based on the local or systemic application of a photosensitive compound—the photosensitizer, which is highly accumulated in pathological tissues. The activation processes leading to the selective destruction of the inappropriate cells is initiated when the photosensitizer molecules absorb the light of the appropriate wavelength. PDT achieves the treatment by causing a series of processes such as apoptosis, autophagy, necrosis and inducing local inflammation. Although normal tissues also can absorb photosensitizers, overly proliferating cells, such as cancer cells, tend to accumulate more photosensitizers. The photosensitizers can be metabolized rapidly in normal tissues [11, 22]. Although HPD-PDT has already proved its usefulness in the treatment of tumors, there are few studies on the effect of PDT on SCLC and bronchiolar epithelium, and the fundamental mechanisms of its effects are still incompletely understood.

The results of this study showed that HPD alone or 630 nm laser irradiation had no obvious inhibitory effects on H446 cells and BEAS-2B cells. When HPD concentration was 15 μg/mL and laser intensity reached 50 MW/cm^2^, the viability of H446 cells and BEAS-2B cells was decreased significantly with the result of 13.96% and 24.23%, and the viability in H446 was significantly lower than in BEAS-2B cells. Continuing to increase the concentration of HPD or the energy densities of 630 nm laser did not significantly increase the severity of growth inhibition. It means that when the photosensitizer absorbed by the cell reaches a certain amount will approach saturation. We confirmed that the optimum dose was 15 g/mL HPD and 50 MW/cm^2^ irradiation. Apoptotic cells were seen in HPD-PDT groups of both H446 cells and BEAS-2B cells and increased with the increase of HPD concentration. The apoptosis of H446 cells and BEAS-2B cells were significantly increased with the apoptosis rates of (46.73 ± 1.38) % and (28.56 ± 1.80) % after being treated with 15 μg/mL HPD and a light energy density of 50 MW/cm^2^, the apoptosis rate of HPD-PDT group in H446 was significantly lower than in BEAS-2B cells. According to the results above, we found that HPD-PDT can induce apoptosis and inhibit invasion of H446 cells and BEAS-2B cells, and the inhibitory effect of HPD-PDT on cells depends on the concentration of HPD and light intensity within a certain range. The inhibitory effect of HPD-PDT on H446 cells is more obvious than on BEAS-2B cells.

Apoptosis is an evolutionarily conserved form of programmed cell death. Evasion of apoptosis is one of the major hallmarks of cancer. It is biochemically and morphologically discrete form of cell death that exhibits major interaction in tissue homeostasis, growth and immune response [23]. Bcl-2 family proteins play an essential role in regulation of the apoptotic pathway and they are divided into pro-apoptotic Bcl-2 family proteins such as Bax, and anti-apoptotic Bcl-2 family proteins such as Bcl-2 [24, 25]. The ratio of the two determines whether cells can receive the apoptotic signal. Bax plays its role in promoting apoptosis by inhibiting the activity of Bcl-2 by forming heterodimers with Bcl-2 [26]. Bcl-2 overexpression is commonly associated with various cancers including breast cancer, prostate cancer, B-cell lymphomas and colorectal adenocarcinomas, etc. Thus, Bcl-2 is a novel anti-cancer target attracting medicinal chemists across the globe [27]. Previous experiments by Guo et al. have shown that PDT leads to the down-regulation of Bcl-2 expression and the up-regulation of Bax expression [28]. In this study, results showed that the relative mRNA expression levels of Bax were significantly increased in H446 cells and BEAS-2B cells after HPD-PDT, and the relative mRNA expression levels of Bcl-2 were significantly decreased in H446 cells and BEAS-2B cells after HPD-PDT. It indicated that HPD-PDT may induce apoptosis by decreasing the Bcl-2/Bax ratio, which is consistent with the results of Guo et al.

Caspases are cysteine-dependent aspartate-specific proteases, involved in the process of apoptosis [29]. Caspase-9 as the apoptotic initiator protease of the intrinsic or mitochondrial apoptotic pathway, is activated at multi-protein activation platforms. Previous studies revealed that the activation of Bcl-2 family members Bax and Bak can cause the release of cytochrome c and other pro-apoptotic proteins. Cytochrome c can bind to Apaf-1 to generate apoptotic bodies, and the activation of Caspase-9 occurred. Activated Caspase 9 can directly cleave and activate Caspase-3 and Caspase-7, accelerating cell apoptosis [30–32]. Our results showed that the relative mRNA expression levels of Bax were significantly increased in H446 cells and BEAS-2B cells after HPD-PDT, and the relative mRNA expression levels of Bcl-2 were significantly decreased in H446 cells and BEAS-2B cells after HPD-PDT.

In summary, HPD-PDT can promote apoptosis and inhibit invasion of small cell lung cancer H446 cells and bronchial epithelial BEAS-2B cells. The detailed signaling cascades and underlying molecular mechanisms remain unclear. The apoptosis may be related to up-regulating the expression levels of Bax and Caspase-9 mRNA and down-regulating the expression levels of Bcl-2 mRNA. The mechanisms involved need to be explored in future research.

## References

[1] Chen W et al (2016) Cancer statistics in China, 2015. CA Cancer J Clin 66(2):115–132 26808342 10.3322/caac.21338

[2] Bade BC, Dela Cruz CS (2020) Lung Cancer Epidemiology, Etiology, and Prevention. Clin Chest Med 41(1):1–24 32008623 10.1016/j.ccm.2019.10.001

[3] Mao Y et al (2016) Epidemiology of Lung Cancer. Surg Oncol Clin N Am 25(3):439–445 27261907 10.1016/j.soc.2016.02.001

[4] Collins LG et al (2007) Lung cancer: diagnosis and management. Am Fam Physician 75(1):56–63 17225705

[5] Kalemkerian GP et al (2013) Small cell lung cancer. J Natl Compr Canc Netw 11(1):78–98 23307984 10.6004/jnccn.2013.0011 PMC3715060

[6] Kalemkerian GP, Schneider BJ (2017) Advances in Small Cell Lung Cancer. Hematol Oncol Clin North Am 31(1):143–156 27912830 10.1016/j.hoc.2016.08.005

[7] Senapathy GJ, George BP, Abrahamse H (2020) Enhancement of Phthalocyanine Mediated Photodynamic Therapy by Catechin on Lung Cancer Cells. Molecules 25(21):4874 33105655 10.3390/molecules25214874 PMC7659931

[8] Byers LA, Rudin CM (2015) Small cell lung cancer: where do we go from here? Cancer 121(5):664–672 25336398 10.1002/cncr.29098 PMC5497465

[9] Dolmans DE, Fukumura D, Jain RK (2003) Photodynamic therapy for cancer. Nat Rev Cancer 3(5):380–387 12724736 10.1038/nrc1071

[10] Agostinis P et al (2011) Photodynamic therapy of cancer: an update. CA Cancer J Clin 61(4):250–281 21617154 10.3322/caac.20114 PMC3209659

[11] Kwiatkowski S et al (2018) Photodynamic therapy - mechanisms, photosensitizers and combinations. Biomed Pharmacother 106:1098–1107 30119176 10.1016/j.biopha.2018.07.049

[12] Bown SG et al (2002) Photodynamic therapy for cancer of the pancreas. Gut 50(4):549–557 11889078 10.1136/gut.50.4.549 PMC1773165

[13] Dos Santos AF et al (2017) Methylene blue photodynamic therapy induces selective and massive cell death in human breast cancer cells 17(1):194 10.1186/s12885-017-3179-7 PMC5353937 28298203

[14] Fargnoli MC, Peris K (2015) Photodynamic therapy for basal cell carcinoma. Future Oncol 11(22):2991–2996 26550910 10.2217/fon.15.208

[15] Inoue K (2017) 5-Aminolevulinic acid-mediated photodynamic therapy for bladder cancer. Int J Urol 24(2):97–101 28191719 10.1111/iju.13291

[16] Hodgkinson N, Kruger CA, Abrahamse H (2017) Targeted photodynamic therapy as potential treatment modality for the eradication of colon cancer and colon cancer stem cells. Tumour Biol 39(10):1010428317734691 28990490 10.1177/1010428317734691

[17] Kelly JF, Snell ME, Berenbaum MC (1975) Photodynamic destruction of human bladder carcinoma. Br J Cancer 31(2):237–244 1164470 10.1038/bjc.1975.30 PMC2009375

[18] Yin H, Yu Y (2019) Identification of the targets of hematoporphyrin derivative in lung adenocarcinoma using integrated network analysis. Biol Res 52(1):4 30717818 10.1186/s40659-019-0213-z PMC6360726

[19] Wang Y et al (2016) A photodynamic therapy combined with topical 5-aminolevulinic acid and systemic hematoporphyrin derivative is more efficient but less phototoxic for cancer. J Cancer Res Clin Oncol 142(4):813–821 26581214 10.1007/s00432-015-2066-3 PMC11819051

[20] Lin C et al (2020) The study of killing effect and inducing apoptosis of 630-nm laser on lung adenocarcinoma A549 cells mediated by hematoporphyrin derivatives in vitro. Lasers Med Sci 35(1):71–78 31049741 10.1007/s10103-019-02794-5

[21] Manyak MJ et al (1988) Photodynamic therapy. J Clin Oncol 6(2):380–391 2963095 10.1200/JCO.1988.6.2.380

[22] Dougherty TJ et al (1998) Photodynamic therapy. J Natl Cancer Inst 90(12):889–905 9637138 10.1093/jnci/90.12.889 PMC4592754

[23] Martinou JC, Youle RJ (2011) Mitochondria in apoptosis: Bcl-2 family members and mitochondrial dynamics. Dev Cell 21(1):92–101 21763611 10.1016/j.devcel.2011.06.017 PMC3156409

[24] Shangary S, Johnson DE (2002) Peptides derived from BH3 domains of Bcl-2 family members: a comparative analysis of inhibition of Bcl-2, Bcl-x(L) and Bax oligomerization, induction of cytochrome c release, and activation of cell death. Biochemistry 41(30):9485–9495 12135371 10.1021/bi025605h

[25] Oltersdorf T et al (2005) An inhibitor of Bcl-2 family proteins induces regression of solid tumours. Nature 435(7042):677–681 15902208 10.1038/nature03579

[26] Yang F et al (2020) Stoichiometry and regulation network of Bcl-2 family complexes quantified by live-cell FRET assay. Cell Mol Life Sci 77(12):2387–2406 31492967 10.1007/s00018-019-03286-z PMC11104934

[27] Suvarna V, Singh V, Murahari M (2019) Current overview on the clinical update of Bcl-2 anti-apoptotic inhibitors for cancer therapy. Eur J Pharmacol 862:172655 31494078 10.1016/j.ejphar.2019.172655

[28] Guo Q et al (2016) 5-Aminolevulinic acid photodynamic therapy in human cervical cancer via the activation of microRNA-143 and suppression of the Bcl-2/Bax signaling pathway. Mol Med Rep 14(1):544–550 27177038 10.3892/mmr.2016.5248

[29] Kim B, Srivastava SK, Kim SH (2015) Caspase-9 as a therapeutic target for treating cancer. Expert Opin Ther Targets 19(1):113–127 25256701 10.1517/14728222.2014.961425

[30] Zhang TM (2019) TRIAP1 Inhibition Activates the Cytochrome c/Apaf-1/Caspase-9 Signaling Pathway to Enhance Human Ovarian Cancer Sensitivity to Cisplatin. Chemotherapy 64(3):119–128 31661694 10.1159/000501633

[31] Singla N, Dhawan DK (2015) Zinc down regulates Apaf-1-dependent Bax/Bcl-2 mediated caspases activation during aluminium induced neurotoxicity. Biometals 28(1):61–73 25381639 10.1007/s10534-014-9803-y

[32] Park HH (2012) Structural features of caspase-activating complexes. Int J Mol Sci 13(4):4807–4818 22606010 10.3390/ijms13044807 PMC3344246

